# Proteomic Alterations in Aqueous Humor From Patients With Primary Open Angle Glaucoma

**DOI:** 10.1167/iovs.17-23434

**Published:** 2018-05

**Authors:** Shruti Sharma, Kathryn E. Bollinger, Sai Karthik Kodeboyina, Wenbo Zhi, Jordan Patton, Shan Bai, Blake Edwards, Lane Ulrich, David Bogorad, Ashok Sharma

**Affiliations:** 1 Center for Biotechnology and Genomic Medicine, Augusta University, Augusta, Georgia, United States; 2 Department of Ophthalmology, Augusta University, Augusta, Georgia, United States; 3 James & Jean Culver Vision Discovery Institute, Augusta University, Augusta, Georgia, United States; 4 Department of Population Health Sciences, Augusta University, Augusta, Georgia, United States

**Keywords:** aqueous humor, glaucoma, mass spectrometry, proteomics

## Abstract

**Purpose:**

Primary open angle glaucoma (POAG) is the most prevalent form of glaucoma, accounting for approximately 90% of all cases. The aqueous humor (AH), a biological fluid in the anterior and posterior chambers of the eye, is involved in a multitude of functions including the maintenance of IOP and ocular homeostasis. This fluid is very close to the pathologic site and is also known to have a significant role in glaucoma pathogenesis. The purpose of this study was to identify proteomic alterations in AH from patients with POAG.

**Methods:**

AH samples were extracted from 47 patients undergoing cataract surgery (controls: *n* = 32; POAG: *n* = 15). Proteomic analysis of the digested samples was accomplished by liquid-chromatography-mass spectrometry. The identified proteins were evaluated using a variety of statistical and bioinformatics methods.

**Results:**

A total of 33 proteins were significantly altered in POAG subjects compared with the controls. The most abundant proteins in POAG subjects are IGKC (13.56-fold), ITIH4 (4.1-fold), APOC3 (3.36-fold), IDH3A (3.11-fold), LOC105369216 (2.98-fold). SERPINF2 (2.94-fold), NPC2 (2.88-fold), SUCLG2 (2.70-fold), KIAA0100 (2.29-fold), CNOT4 (2.23-fold), AQP4 (2.11-fold), COL18A1 (2.08-fold), NWD1 (2.07-fold), and TMEM120B (2.06-fold). A significant increasing trend in the odds ratios of having POAG was observed with increased levels of these proteins.

**Conclusion:**

Proteins identified in this study are implicated in signaling, glycosylation, immune response, molecular transport, and lipid metabolism. The identified candidate proteins may be potential biomarkers associated with POAG development and may lead to more insight in understanding the mechanisms underlying the pathogenesis of this disease.

Glaucoma is the leading cause of irreversible blindness worldwide. By 2020, approximately 76 million people are expected to develop glaucoma, making it a major global health problem.^1^ Glaucoma, if untreated, leads to irreversible blindness caused by a progressive optic neuropathy with retinal ganglion cell (RGC) death, which results in a characteristic visual field loss. Precise molecular mechanisms of the RGC apoptosis and loss of axons at the optic nerve are not known. Glaucoma can broadly be classified as either angle closure glaucoma (ACG) or open angle glaucoma (OAG). In ACG, the anterior chamber angle is acutely or chronically closed by the iris moving forward, obstructing aqueous outflow and can clinically be seen as a closed angle on gonioscopy.^2^,^3^ In OAG, the angle remains open on gonioscopy, but outflow is obstructed by various other mechanisms at the level of the trabecular meshwork (TM). OAG can be further subdivided into secondary, where an underlying cause (steroid induced, pigmentary, pseudoexfoliation, etc.) is identified, or primary, with no underlying cause. By far, the most prevalent form of the disease is primary open angle glaucoma (POAG), which accounts for 90% of all cases of glaucoma.^4^ Clinically, POAG presents itself with evidence of optic disc or retinal nerve fiber layer (RNFL) structural abnormalities (such as increased cup to disc ratio) and characteristic visual field loss.^5^ Elevated IOP is a major risk factor for glaucoma, but the disease can develop in patients with normal intraocular pressure. In addition, many patients progressively suffer vision loss despite IOP-lowering treatment. Although additional risk factors including age, ethnicity, family history, and central corneal thickness have been identified, early detection of glaucoma remains a challenge.

The aqueous humor (AH) is a clear biological fluid that fills the anterior segment of the eye from the anterior vitreous face forward to the cornea. Circulating forward from the ciliary epithelium, it bathes the lens, passes through the pupil, across the iris, and exits through the TM. On this path through the eye, it becomes invested with both normal and pathologic proteins. AH is involved in a multitude of functions, perhaps the most important of which is the maintenance of IOP.^6^ Although some proteins in the AH are represented in patient serum, this is not universal. Some proteins in the AH are not present in serum and thus must be secreted from anterior segment tissues^7^ rather than being an ultrafiltrate from ciliary body capillary beds. Although the concentration of proteins in AH is nominal (120 to 500 ng/μL), its role in ocular disorders has been gaining prominence.^7^–^17^ AH has also been shown to be involved in myocilin secretion (a protein associated with the development of glaucoma) from vesicle-like structures within the cytoplasm of TM cells.^18^ Elevated TGFβ2 is observed in the AH of POAG patients,^19^ which in turn regulates various signaling pathways of the extracellular matrix (ECM) components.^19^,^20^ This fluid is very close to the pathologic site and is known to have a significant role in glaucoma pathogenesis. Therefore, in this study, to discover POAG-related alterations in the AH protein content, proteomic analysis was performed using label-free mass spectrometry that provides a semiquantitative analysis of peptides to quantitate all proteins present in a sample.

## Materials and Methods

### Subjects

We analyzed proteomes of human AH from 47 patients undergoing cataract eye surgery in the Medical College of Georgia at Augusta University. Of these, 32 were control subjects and 15 patients carried a clinically established diagnosis of POAG. Clinical diagnosis of POAG was made by one of three staff ophthalmologists (KEB, LU, or DB). Diagnosis was based on optic nerve appearance and ancillary testing, as indicated, dependent on clinical findings. Optic nerve examination characteristics indicative of glaucoma included progressive cupping, thinning of the neuroretinal rim, notch formation, and disc hemorrhage. Ancillary tests included visual field (Humphrey visual field analyzer; Carl Zeiss Meditec, Dublin, CA, USA), fundus photographs of optic nerve (Zeiss 450+ fundus camera; Carl Zeiss Meditec), and spectral-domain optical coherence tomography (Spectralis OCT; Heidelberg Engineering, Heidelberg, Germany). Elevated IOP was not used as a diagnostic criterion.

All 32 control subjects were undergoing cataract surgery by phacoemulsification. Of the 15 glaucoma patients, 13 were undergoing cataract surgery at time of collection and 2 underwent aqueous tube shunt surgery. The AH was collected using a paracentesis incision, which was made through clear cornea. Using this technique, the lens was not violated, and no bleeding occurred. Written informed consents were obtained from all study participants. The study was approved by Institutional Review Boards at Augusta University, Augusta, GA. A chart review was conducted for all glaucoma and control subjects that included an assessment of age, race, sex, body mass index (BMI), tobacco use, presence of collagen vascular disease, cerebrovascular disease, hypertension, and diabetes. Glaucoma-specific characteristics were also recorded including clinical cup to disc ratio and IOP (Table 1).

**Table 1:** Baseline Characteristics of Normal Controls and POAG Subjects

**Patient Characteristics**	**Control**	**POAG**	***P*** **Value**
Subjects, *n*	32	15	NA
Female/male	20/12	11/4	0.688
Age, y	65.8 ± 9.1	65.1 ± 14.3	0.855
Age range, y	48–82	35–83	NA
Race: African American/ Caucasian	15/17	8/7	0.920
BMI	29.8 ± 5.5	32.2 ± 7.8	0.387
Hypertension, N/Y	2/30	2/12	0.747
Smoking, N/Y	23/4	13/2	1.000
Cardiovascular disease, N/Y	28/4	12/1	1.000
Cerebrovascular disease, N/Y	29/3	13/ 0	0.628
Collagen vascular disease, N/Y	32/0	13/0	NA
Diabetes, N/Y	19/13	8/5	1.000
Cup/disc ratio	0.27	0.62*	3 × 10^−4^
IOP	13.8 ± 2.6	16.1 ± 6.9	0.223

NA, not available.

* < 0.05.

### Sample Preparation

For this study, 20- to 60-μL AH samples were first lyophilized and then reconstituted in 30 μL 8 M urea in 50 mM Tris-HCl (pH 8). Reduction and alkylation of the cysteine residues were then performed with 20 mM dithiothreitol and 55 mM iodoacetamide, respectively, followed by adding 240 μL 50 mM ammonium bicarbonate buffer to reduce the urea concentration to below 1 M. Protein concentration was measured using a Bradford assay kit according to the manufacturer's instruction (Pierce, Rockford, IL, USA). Trypsin (Pierce) was then added at a 1:20 ratio (w/w) to perform protein digestion at 37°C overnight.

### Liquid Chromatography-Mass Spectrometry Analysis

All 47 samples were processed individually for liquid-chromatography-mass spectrometry (LC-MS/MS) analysis. Digested AH samples were first cleaned using a C18 spin plate (Nest Group, Southborough, MA, USA) and then analyzed using the Orbitrap Fusion tribrid mass spectrometer (Thermo Scientific, Waltham, MA, USA) coupled with an Ultimate 3000 nano-UPLC system (Thermo Scientific). Six microliters of reconstituted peptide was trapped and washed on a Pepmap100 C18 trap (5 μm, 0.3 × 5 mm) at 20 μL/min using 2% acetonitrile in water (with 0.1% formic acid) for 10 minutes and then separated on a Pepman100 RSLC C18 column (2.0 μm, 75-μm × 150-mm) using a gradient of 2% to 40% acetonitrile with 0.1% formic acid over 120 minutes at a flow rate of 300 nL/min and a column temperature of 40°C. Eluted peptides were introduced into the Orbitrap Fusion MS via nano-electrospray ionization source with a temperature of 275°C and spray voltage of 2000 V and analyzed by data-dependent acquisition in positive mode using the Orbitrap MS analyzer for precursor scan at 120,000 FWHM from 300 to 1500 m/z and ion-trap MS analyzer for MS/MS scans in top speed mode (2-second cycle time) with dynamic exclusion settings (repeat count 1, repeat duration 15 seconds, and exclusion duration 30 seconds). Collision-induced dissociation (CID) was used as a fragmentation method with a normalized collision energy of 30%.

### Protein Identification and Quantification

Raw MS data were processed using the Proteome Discoverer (v1.4; Thermo Scientific) and submitted for SequestHT search against the Uniprot human protein database (8/26/2015, 114,210 entries). SequestHT search parameters were 10 ppm precursor and 0.6 Da product ion tolerance, with static carbidomethylation (+57.021 Da) for cysteine and dynamic oxidation (+15.995 Da) for methionine and dynamic phosphorylation (+79.966 Da) for serine, threonine, and tyrosine. The Percolator peptide spectrum matching (PSM) validator algorithm was used for PSM validation. Proteins that contained similar peptides and could not be differentiated based on MS/MS analysis alone were grouped to satisfy the principles of parsimony. Proteins sharing significant peptide evidence were grouped into clusters. The protein report comprising the identities and spectrum counts (number of PSM) for each protein was then exported as a semiquantitative measure for relative protein expression levels in all samples. The PSM count for each protein was normalized against AH sample volume to correct for starting volume differences.

### Statistical Analyses

PSM count data from a total of 401 proteins obtained from previous step were subjected to statistical analyses. All statistical analyses were performed using the R Project for Statistical Computing (version 3.2.5). The protein levels were log2 transformed prior to all statistical analyses to achieve normal distribution. To examine the relationship between POAG status and protein levels, we analyzed the data using multivariate logistic regression analysis, adjusting for important covariates including, age, sex, race, IOP, and presence of hypertension. The ORs of the proteins were calculated with 95% CI, and their significance was assessed through *P* values. The area under the curve (AUC) of the receiver operating characteristic (ROC) curves was calculated to evaluate their utility as POAG biomarkers. In addition, the risk of PAOG with increased protein levels was evaluated using protein quartiles. The subjects were divided into four quartiles based on protein levels, and the first quartile was used as a reference. The ORs of having POAG was calculated for the remaining quartiles to identify the proteins associated with progression of POAG.

In an attempt to identify the biological processes and pathways affected by the altered proteins, functional annotation was performed using The Database for Annotation, Visualization and Integrated Discovery (DAVID) Bioinformatics Resources (v6.8) and Ingenuity Pathway Analysis (IPA). Network analysis was also performed using IPA to search for defined interactions between these 33 proteins associated with POAG.

## Results

The demographics of the subjects used in this study are presented in the Table 1. There was no significant difference between POAG and control groups for their age, race, BMI, sex, hypertension, smoking history, and presence of cardiovascular disease, cerebrovascular disease, collagen vascular disease, and diabetes. The mean cup/disc ratio was significantly higher in the POAG compared with controls (0.62 vs. 0.27; *P* = 3 × 10^−4^), whereas mean IOP was slightly higher in POAG (16.1 vs. 13.8; *P* = 0.223), but it was not statistically significant.

### Proteomic Changes in AH From POAG Patients

**Figure 1:**
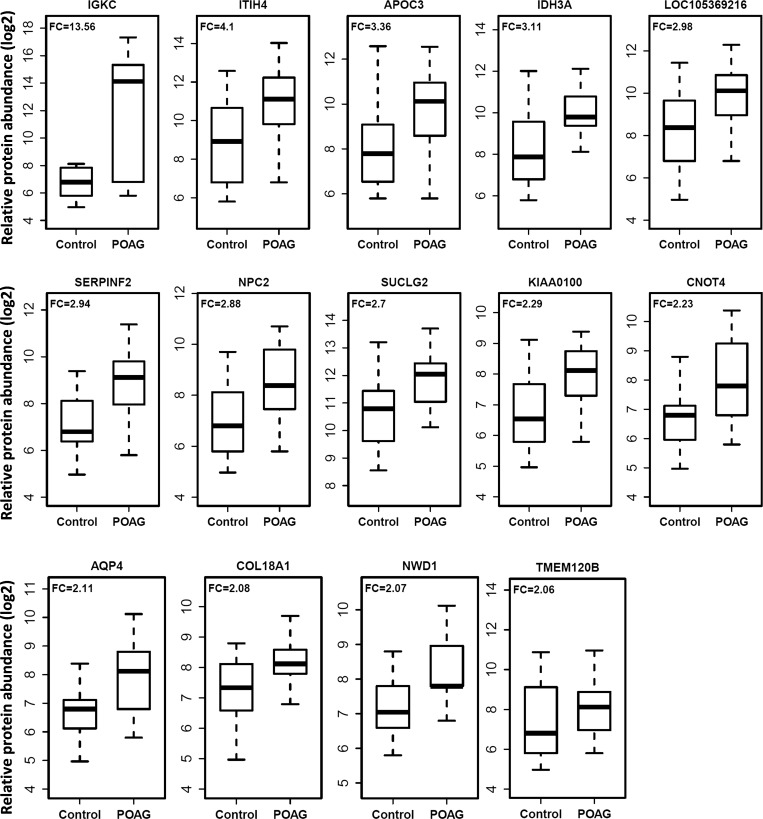
Box plots showing distribution of relative protein abundance in the AH from subjects with POAG (n = 15) and control subjects without POAG undergoing cataract surgery (n = 32). FC represents the ratios between the mean levels in POAG and control groups for each protein.

Multivariate analyses yielded a total of 33 AH proteins that were significantly associated with POAG. The ORs and fold-change values of these 33 proteins are presented in Table 2. The highest increase was found in the Ig κ chain C region protein (IGKC; 13.56-fold). Other top proteins that are most elevated in the AH of glaucoma patients are Inter-α-trypsin inhibitor heavy chain 4 (ITIH4; 4.10-fold), Apolipoprotein C-III (APOC3; 3.36-fold), Isocitrate dehydrogenase [NAD] subunit α (IDH3A; 3.11-fold), cDNA FLJ42083 fis, clone TCERX2000613 (LOC105369216; 2.98-fold), Serine/cysteine proteinase inhibitor clade F (SERPINF2; 2.94-fold), Niemann-pick disease, type C2 (NPC2; 2.88-fold), Succinate-CoA ligase subunit β (SUCLG2; 2.70-fold), KIAA0100 ( 2.29-fold), CCR4-NOT transcription complex subunit 4 (CNOT4; 2.23-fold), Aquaporin 4 (AQP4; 2.11-fold), Collagen, type XVIII, α 1 (COL18A1; 2.08-fold), and NACHT and WD repeat domain containing 1 (NWD1; 2.07-fold) (Table 2). The boxplots showing the distribution of the top 14 proteins (greater than twofold) are presented in Figure 1.

**Table 2:** AH Proteins Significantly Altered in Subjects With POAG

**S. No.**	**Protein Name**	**Gene Symbol**	**UniProt ID**	**OR (95% CI)**	***P*** **Value**	**Fold Change**
1	Ig κ chain C region	*IGKC*	A0A087X130	1.31 (1.065–1.609)	0.0104	13.56
2	Inter-α-Trypsin Inhibitor heavy chain4	*ITIH4*	B7Z544	1.64 (1.065–2.521)	0.0248	4.10
3	Apolipoprotein C-III	*APOC3*	A3KPE2	1.90 (1.181–3.048)	0.0081	3.36
4	Isocitrate dehydrogenase [NAD] subunit α	*IDH3A*	H0YLI6	1.98 (1.141–3.434)	0.0152	3.11
5	cDNA FLJ42083 fis, clone TCERX2000613	*LOC105369216*	Q6ZVM5	1.90 (1.033–3.508)	0.0391	2.98
6	Serine/cysteine proteinase inhibitor clade F	*SERPINF2*	C9JPV4	1.55 (1.046–2.304)	0.0292	2.94
7	Niemann-Pick disease, type C2	*NPC2*	G3V2V8	1.99 (1.150–3.452)	0.0139	2.88
8	Succinate-CoA ligase subunit β	*SUCLG2*	B4DRV2	2.57 (1.147–5.752)	0.0218	2.70
9	KIAA0100	*KIAA0100*	K7EQ86	2.62 (1.255–5.472)	0.0103	2.29
10	CCR4-NOT transcription complex subunit 4	*CNOT4*	B3KQ99	3.80 (1.421–10.171)	0.0078	2.23
11	Aquaporin 4	*AQP4*	L0R6C7	3.63 (1.381–9.563)	0.0090	2.11
12	Collagen, type XVIII, α 1	*COL18A1*	Q8NG19	2.64 (1.030–6.756)	0.0432	2.08
13	NACHT and WD repeat domain containing 1	*NWD1*	F8W0U9	2.09 (1.069–4.068)	0.0312	2.07
14	Transmembrane protein 120B	*TMEM120B*	H0YG77	1.59 (1.025–2.456)	0.0383	2.06
15	Leucine rich repeat containing 34	*LRRC34*	G3V115	2.40 (1.143–5.028)	0.0207	1.98
16	IgGFc-binding protein	*FCGBP*	A0A087WUZ2	2.10 (1.114–3.966)	0.0219	1.92
17	Na+/K+ Transporting ATPase Interacting 2	*NKAIN2*	B3KNZ0	2.07 (1.064–4.007)	0.0321	1.92
18	Solute Carrier Family 35 Member C2	*SLC35C2*	B7Z6R4	2.29 (1.095–4.798)	0.0278	1.89
19	Tetraspanin 14	*TSPAN14*	Q8N2P5	2.39 (1.107–5.151)	0.0265	1.88
20	Family With Sequence Similarity 171, Member B	*FAM171B*	A0A087WU95	2.28 (1.092–4.758)	0.0282	1.87
21	Fetuin-B	*FETUB*	F8WEP7	2.22 (1.079–4.575)	0.0303	1.86
22	LDL receptor-related protein-5	*LRP5*	E9PHY1	2.25 (1.069–4.725)	0.0327	1.83
23	Fibrinogen β chain	*FGB*	D6REL8	1.93 (1.041–3.576)	0.0368	1.82
24	Hydroxysteroid (17-β) Dehydrogenase 10	*HSD17B10*	Q5H928	2.43 (1.070–5.511)	0.0339	1.80
25	Phosphatidylinositol glycan, class C	*PIGC*	A0A024R900	2.06 (1.012–4.190)	0.0464	1.78
26	Protein cornichon homolog 1	*CNIH1*	G3V5P8	2.66 (1.173–6.048)	0.0192	1.75
27	Torsin family 3, member A	*TOR3A*	A0A024R943	2.49 (1.142–5.448)	0.0218	1.73
28	Mediator complex subunit MED23	*MED23*	B9TX53	2.26 (1.113–4.590)	0.0240	1.68
29	Succinate dehydrogenase assembly factor 2	*SDHAF2*	M0QY91	2.09 (1.089–4.004)	0.0267	1.67
30	Protein COMMD3-BMI1	*COMMD3-BMI1*	Q5T8Z1	2.05 (1.016–4.149)	0.0452	1.64
31	Inositol polyphosphate 1-phosphatase	*INPP1*	C9J128	2.12 (1.044–4.302)	0.0376	1.59
32	cDNA FLJ57526	N/A	B4DLF2	2.00 (1.036–3.839)	0.0388	1.54
33	Rheumatoid factor D5 light chain	*V-kappa-3*	A0N5G5	2.36 (1.030–5.406)	0.0424	1.46

### Evaluation of Altered AH Proteins for POAG Biomarkers

**Figure 2:**
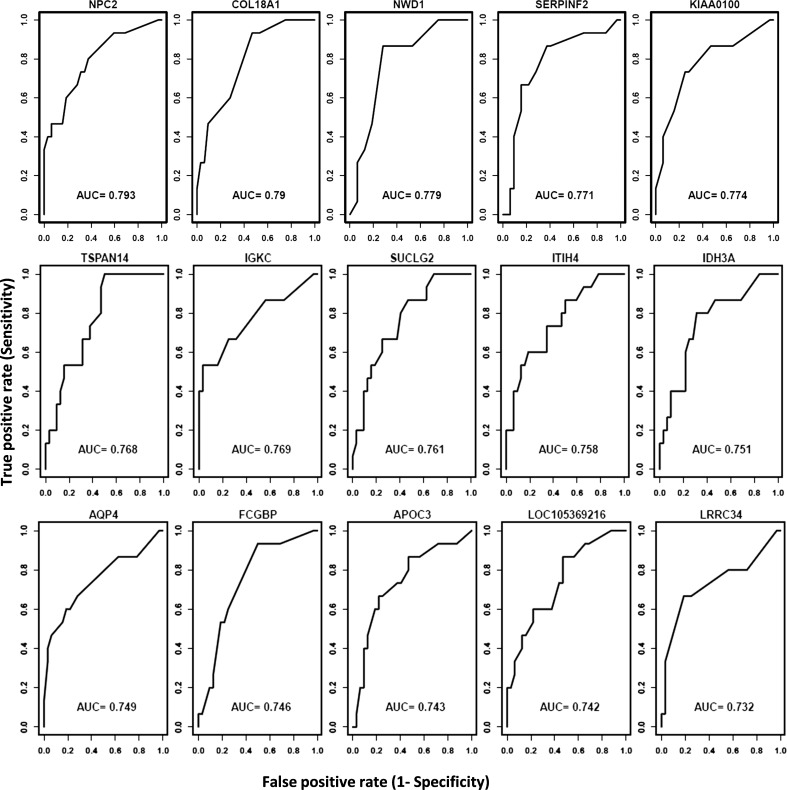
ROC curves of the top more abundant proteins in POAG. AUC values represent the significance of the AH proteins as POAG biomarkers.

The utility of the proteins as POAG biomarkers was evaluated using ROC analysis. The AH proteins with the highest AUC values are shown in Figure 2. Several proteins have excellent biomarker potential to separate controls and glaucoma patients, including NPC2 (AUC = 0.793), COL18A1 (AUC = 0.790), NWD1 (AUC = 0.779), SERPINF2 (AUC = 0.771), TSPAN14 (AUC = 0.768), IGKC (AUC = 0.769), SUCGL2 (AUC = 0.761), ITIH4 (AUC = 0.758), and IDH3A (AUC = 0.751).

### Risk Analysis Using Quartiles

**Figure 3:**
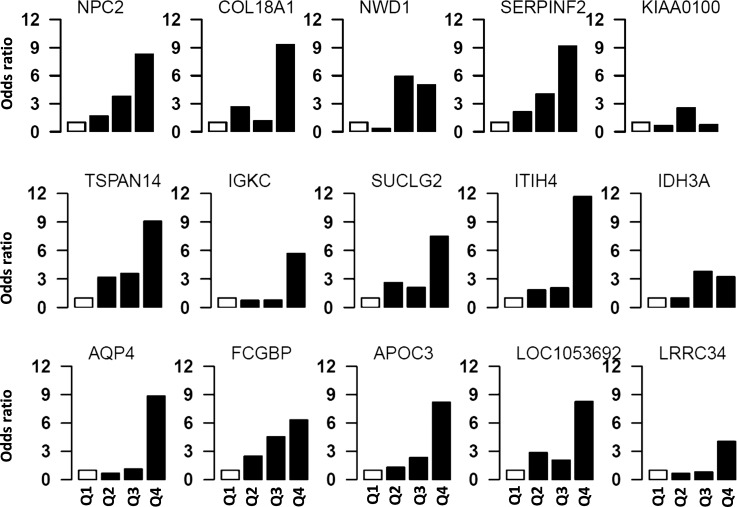
The risk of POAG in subjects with different protein quartiles. Individual protein levels were used to assess the OR of having POAG at different protein levels. All subjects were divided into four quartiles (Q1–Q4) based on protein levels. The first quartile (Q1) was used as reference, and ORs of having POAG was calculated for upper three quartiles. Analyses using quartiles revealed that risk of POAG increases with increasing levels of these proteins. The open bar represents the first quartile as reference (OR = 1). From left to right, each of the other three solid bars represent second to fourth quartiles (Q2–Q4).

Subjects were divided into four quartiles based on protein levels, and the first quartile was used as a reference. The ORs of having POAG for the remaining three quartiles were calculated in relation to the first quartile. A significant increasing trend in the ORs of having POAG was observed with increased levels of these proteins as shown in Figure 3.

### Bioinformatics Analyses

To discover associations of the 33 significantly altered proteins with biological functional categories and canonical pathways, bioinformatics analyses were performed (Table 3). Significantly enriched functional categories include glycoproteins (*n* = 15, *P* = 1.1 × 10^−3^), signaling pathways proteins (*n* = 15, *P* = 4.1 × 10^−4^), secreted proteins (*n* = 10, *P* = 4.2 × 10^−4^), proteins possessing an N-linked glycosylation site (N-GlcNAc) (*n* = 14, *P* = 1.0 × 10^−3^), and signal peptides (*n* = 13, *P* = 3.6 × 10^−4^). Enriched cellular compartments are extracellular exosome (*n* = 10, *P* = 1.2 × 10^−2^) and extracellular region (*n* = 8, *P* = 3.6 × 10^−3^; Table 3). Negative regulation of endopeptidase activity (*n* = 3, *P* = 6.6 × 10^−4^), platelet degranulation (*n* = 3, *P* = 4.1 × 10^−4^), and cholesterol homeostasis (*n* = 3, *P* = 1.0 × 10^−4^) are significantly enriched ontology terms. The top canonical pathways enriched in these proteins include Farnesoid X receptor (FXR)/retinoid X receptors (RXR) activation (*n* = 4, *P* = 2.5 × 10^−5^), liver X receptors (LXR)/RXR activation (*n* = 3, *P* = 6.0 × 10^−4^), and acute phase response signaling (*n* = 3, *P* = 1.6 × 10^−3^; Table 3).

**Table 3:** Biological Functions and Pathways Significantly Enriched in Proteins Associated With POAG

**Term**	**Count**	***P*** **Value**
Uniprot keywords		
Signaling pathways proteins	15	4.1 × 10^−4^
Glycoprotein	15	1.1 × 10^−3^
Secreted proteins	10	4.2 × 10^−4^
Disease mutation	9	1.2 × 10^−2^
Protease inhibitor	3	8.5 × 10^−4^
Uniprot sequence features		
Glycosylation site: N-linked (N-GlcNAc)	14	1.0 × 10^−3^
Signal peptide	13	3.6 × 10^−4^
Disulfide bond	9	1.8 × 10^−2^
Glycosylation site: O-linked (O-GlcNAc)	3	3.7 × 10^−4^
Cellular compartments		
Extracellular exosome	10	1.2 × 10^−2^
Extracellular region	8	3.6 × 10^−3^
Blood microparticle	4	1.1 × 10^−4^
Mitochondrial matrix	4	1.9 × 10^−3^
External side of plasma membrane	3	5.1 × 10^−3^
Biological processes		
Negative regulation of endopeptidase activity	3	6.6 × 10^−4^
Platelet degranulation	3	4.1 × 10^−4^
Cholesterol homeostasis	3	1.0 × 10^−4^
Molecular and cellular functions		
Small molecule biochemistry	8	9.9 × 10^−5^
Lipid metabolism	7	9.9 × 10^−5^
Molecular transport	7	9.9 × 10^−5^
Cell death and survival	6	7.4 × 10^−4^
Vitamin and mineral metabolism	5	4.0 × 10^−4^
Canonical pathways		
FXR/RXR activation	4	2.5 × 10^−5^
LXR/RXR activation	3	6.0 × 10^−4^
Acute phase response signaling	3	1.6 × 10^−3^
Intrinsic prothrombin activation pathway	2	1.5 × 10^−3^
Coagulation pathway	2	1.0 × 10^−3^

**Figure 4:**
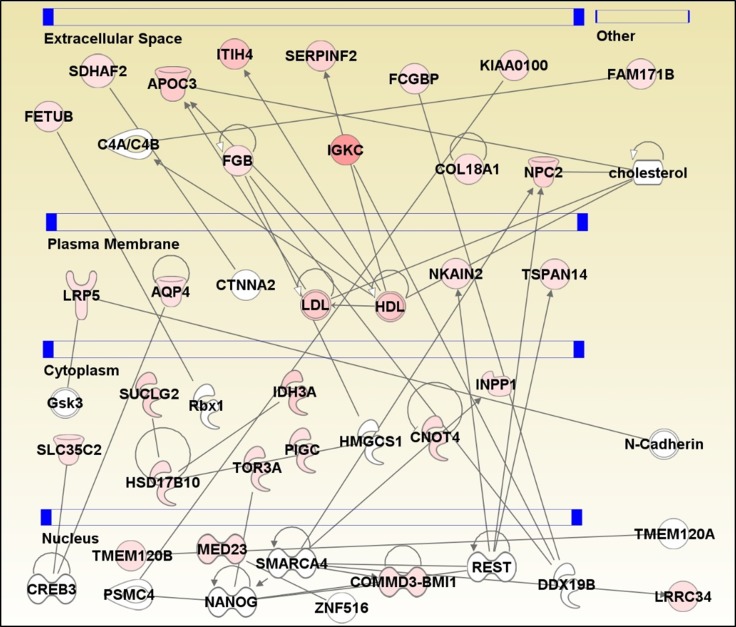
Network analysis using IPA was performed to search for defined interactions between 33 proteins associated with POAG. Each protein is represented as a node, and an edge represents an interaction between two nodes. Red nodes indicate more abundant proteins, whereas white nodes are proteins not significantly altered in POAG but are required for network stability. A solid line represents direct functional interaction. An arrow indicates action of a protein product on a target. Circular arrow indicates a self-referential relationship. Proteins are separated based on their cellular compartments.

IPA software was used to generate a protein interaction network based on known interactions (Fig. 4). The proteins in the network are highly enriched in lipid metabolism, small molecule biochemistry, and molecular transport. LDL and HDL are two key nodes in the network, and APOC3, NPC2, LDL, and HDL are connected to cholesterol, strengthening the evidence that lipid metabolism pathway proteins are upregulated in AH from glaucoma patients. IPA analysis also revealed that a large number of altered proteins in AH including FETUB, SDHAF2, APOC3, FGB, ITIH4, SERPINF2, IGKC, FCGBP, COL18A1, NPC2, and KIAA0100 belong to the extracellular space. Repressor element 1 silencing transcription factor (REST) and ATP-dependent chromatin remodeler (SMARCA4) transcription factors are upstream regulators of several key proteins including COMMD3-BMI1, INPP1, LRRC34, NKAIN2, NPC2, and TSPAN14.

## Discussion

The aim of this study was to discover alterations in the AH proteome during POAG disease pathogenesis. We identified a total of 33 proteins that were significantly altered in the POAG patients compared with controls. Bioinformatics analyses revealed that the altered AH proteins are involved in a variety of cellular functions including signaling, glycosylation, immune response, molecular transport, and lipid metabolism.

The trabecular meshwork is known to have a major role in POAG pathogenesis.^21^ One of the components of TM is the juxtacanalicular tissue, which is composed of glycosaminoglycans and glycoproteins.^21^ Obstruction to the flow of AH stems from structural changes to the juxtacanalicular region, perhaps due to extracellular matrix accumulation and fibrillary elements associated with glycoproteins.^21^ Our analyses revealed that 15 of 33 significantly altered AH proteins in POAG belong to the category of “glycoproteins.”

The top two canonical pathways significantly enriched in proteins altered in POAG are LXR/RXR activation and FXR/RXR activation. The RXRs, LXRs, and FXRs are nuclear receptors that are involved in lipid metabolism, inflammation, and cholesterol conversion to bile acids.^22^,^23^ Network analysis also revealed that a cluster of proteins in the network are also involved in lipid metabolism. In general, our findings are in concordance with evidence of dysregulated lipid metabolism in glaucoma.^22^,^24^

Similar to the findings from Kliuchnikova et al., endopeptidase inhibitor activity and protease inhibitors were significantly enriched in the altered AH proteome.^10^ Regulation of proteolysis is integral to maintaining the transparency of ocular tissues. For example, transparency of the eye may be undermined from the aggregation of proteolytic products acting as antichaperones.^25^ Our bioinformatics analyses revealed that Serpin Family F member 2 (SERPINF2), Fetuin-B (FETUB), and Inter-α-trypsin inhibitor heavy chain H4 (ITIH4) are protease inhibitors involved in negative regulation of endopeptidase activity.

Another interesting finding was that some proteins identified in this study were part of various facets of the immune system. Some of them were associated with immunoglobulin complexes, whereas others were involved in innate immune response and acute phase response signaling. These proteins might play a role in inflammatory processes associated with glaucoma. Immunoglobulin κ constant region (IGKC), immunoglobulin γ Fc binding protein (FCGBP), Tetraspanin 14 (TSPAN14 ), and Inter-α-Trypsin Inhibitor heavy chain4 (ITIH4) are important proteins known to be involved in immunity and inflammation. IGKC is important for the structure of immunoglobulins, which is involved at multiple levels of innate and adaptive immunity. ITIH4 is a type II acute-phase protein involved in inflammatory responses to trauma.^26^

Cell membrane bound proteins including NPC2, KIAA0100, AQP4, and TSPAN14 are associated with transport and trafficking. Niemann Pick Type C2 (NPC2) is important for cell transport of lipids in and out of lysosomes and is implicated in the pathogenesis of Niemann Pick Disease.^27^ Aquaporin 4 (AQP4) is important for cellular water transport across membranes and is associated with various central nervous system disorders.^28^ However, in a study investigating the altered expression of aquaporins in glaucomatous eyes, AQP4 expression was unaltered in the optic nerve fibers of POAG patients.^29^ Tetraspanin-14 (TSPAN14) is important for regulating the maturation and trafficking of transmembrane metalloprotease ADAM10.^30^ Although the exact role of TSPAN14 is yet to be investigated in glaucoma, a member of the tetraspanin family (TSPAN6) was found to be abundant in the TM of POAG patients.^31^

Three cell cycle and development proteins (COL18A1, LRP5, and CNOT4) were also identified. Collagen α-1 (XVIII) chain (COL18A1) is important for development of retinal structure, closing the neural tube, and is also important for ECM organization.^32^ Abnormal retinal angiogenesis and morphology was observed in mice lacking COL18A1.^33^ Furthermore, genome-wide association studies revealed the link between COL18A1 and early-onset glaucoma.^34^ The functional relevance of COL18A1 in glaucoma is yet to be explored; however, there is sufficient evidence illustrating the importance of COL18A1 in eye development, its structural maintenance throughout life, RPE function, and blood vessel formation in the eye.^33^,^35^,^36^ LDL receptor-related protein 5 (LRP5) is an important component of the Wnt-Fzd-LRP5-LRP6 complex that triggers β-catenin signaling, important for development, and is indicated in pathogenesis of familial exudative vitreoretinopathy (FEVR).^37^ In addition, LRP5 is important for sprouting and migration of vascular endothelial cells into deeper layers of the retina, indicating its role in blood vessel morphology and development.^38^ CCR4-NOT transcription complex subunit 4 (CNOT4), which has ubiquitin ligase activity, is involved in the JAK/STAT pathway and is also part of the DNA damage response and arrest of cell cycle through p53.^39^ Two proteins, IDH3A (Isocitrate dehydrogenase [NAD] subunit α) and SUCLG2 (succinate-CoA ligase [GDP-forming] subunit β) are mitochondrial proteins involved in the TCA cycle. Other studies have also shown that several Kreb cycle proteins are involved in the glaucomatous trabecular meshwork.^40^

The limitations of this study include the use of cataract patients undergoing elective surgery as controls, which may affect the results. Collection of AH from healthy subjects is a highly invasive procedure. Also, due to the small volumes of AH obtained, sufficient samples were not available for further validation methods such as Western blotting. Although we can demonstrate a correlation with various proteins and the disease, we cannot prove causality. However, this study provides several proteins and pathways altered in the AH from POAG patients and further studies can be planned to validate these findings and to elucidate the precise molecular mechanisms of these proteins in the pathogenesis of glaucoma.
